# A hybrid feature selection algorithm to determine effective factors in predictive model of success rate for in vitro fertilization/intracytoplasmic sperm injection treatment: A cross-sectional study

**DOI:** 10.18502/ijrm.v21i12.15038

**Published:** 2024-01-25

**Authors:** Ameneh Mehrjerd, Hassan Rezaei, Saeid Eslami, Nayyere Khadem Ghaebi

**Affiliations:** ^1^Department of Computer Sciences, Faculty of Mathematics, Statistics and Computer Sciences, University of Sistan and Baluchestan, Zahedan, Iran.; ^2^Department of Medical Informatics, Faculty of Medicine, Mashhad University of Medical Sciences, Mashhad, Iran.; ^3^Department of Medical Informatics, Academic Medical Center, University of Amsterdam, Amsterdam, the Netherlands.; ^4^Department of Obstetrics and Gynecology, Faculty of Medicine, Mashhad University of Medical Sciences, Mashhad, Iran.

**Keywords:** Machine learning, Feature selection, Infertility treatment, Hesitant fuzzy set.

## Abstract

**Background:**

Previous research has identified key factors affecting in vitro fertilization or intracytoplasmic sperm injection success, yet the lack of a standardized approach for various treatments remains a challenge.

**Objective:**

The objective of this study is to utilize a machine learning approach to identify the principal predictors of success in in vitro fertilization and intracytoplasmic sperm injection treatments.

**Materials and Methods:**

We collected data from 734 individuals at 2 infertility centers in Mashhad, Iran between November 2016 and March 2017. We employed feature selection methods to reduce dimensionality in a random forest model, guided by hesitant fuzzy sets (HFSs). A hybrid approach enhanced predictor identification and accuracy (ACC), as assessed using machine learning metrics such as Matthew's correlation coefficient, runtime, ACC, area under the receiver operating characteristic curve, precision or positive predictive value, recall, and F-Score, demonstrating the effectiveness of combining feature selection methods.

**Results:**

Our hybrid feature selection method excelled with the highest ACC (0.795), area under the receiver operating characteristic curve (0.72), and F-Score (0.8), while selecting only 7 features. These included follicle-stimulation hormone (FSH), 16Cells, FAge, oocytes, quality of transferred embryos (GIII), compact, and unsuccessful.

**Conclusion:**

We introduced HFSs in our novel method to select influential features for predicting infertility success rates. Using a multi-center dataset, HFSs improved feature selection by reducing the number of features based on standard deviation among criteria. Results showed significant differences between pregnant and non-pregnant groups for selected features, including FSH, FAge, 16Cells, oocytes, GIII, and compact. We also found a significant correlation between FAge and fetal heart rate and clinical pregnancy rate, with the highest FSH level (31.87%) observed for doses ranging from 10-13 (mIU/ml).

## 1. Introduction

Infertility prompts couples worldwide to seek medical help for successful conception. Diagnosing its causes and predicting treatment success are essential for guiding interventions and identifying key factors. Several predictive models have been developed using machine learning tools and classification methods to forecast the success rate of infertility treatment (1-4). Identifying crucial factors for predicting infertility treatment success is essential in clinical practice. This challenge is solved using predictive models for infertility treatment. Many predictive models for the success rate of infertility treatment have been presented so far, which have been modeled using machine learning tools and various classification methods (5-8). Feature selection is one of the methods of reducing dimensions to improve the model's performance and determine the essential factors.

Feature selection enhances model performance and identifies essential factors in predictive models. Various techniques have been used in previous studies. Existing studies have employed various predictive models using different feature selection techniques. Some studies used statistical methods, such as Chi-square and student *t* test, to select feature (9), while others used filter-based methods, such as principal component analysis (10). Also, there are studies based on wrapper-based methods, such as forward float selection (11). Another study applied embedded methods such as linear support vector classifier and tree-based for using feature selection process (12).

Furthermore, meta-heuristic algorithms, such as the hill-climbing algorithm (13), were used to select practical features in infertility treatment methods. Also, many models have been proposed to predict the success of therapy, which is to compare the performance of different prediction models without a feature selection method. The collected features are listed based on the expert domain of models (14, 15). A combination of the wrapper, filter, and embedded feature selection methods were used in machine learning. Hesitant fuzzy sets (HFSs) are used to rank methods in determining the similarity between features and output, to improve the performance of the combination (16).

Challenges remain in predictive models for infertility treatment, particularly in feature selection, which is critical for success. Different methods of feature selection in different studies lead to inconsistent results and difficulty in identifying important factors. Using different scoring criteria may not fully represent clinical significance. A comprehensive hybrid approach can improve the accuracy and reliability of predictive models. Previous research has explored single-feature selection techniques, but a comprehensive hybrid approach can improve the accuracy of predictive models. Our primary objective is to address these challenges by proposing a novel hybrid method using HFSs for feature selection in the prediction of in vitro fertilization/intra-cytoplasmic sperm injection (IVF/ICSI) infertility treatment success. This research aims to improve the accuracy and reliability of the prediction model by integrating filter, wrapper, and embedded techniques. The outcome of this research will provide clinicians and medical practitioners with more precise insights into the essential factors contributing to treatment success, guiding personalized interventions, and ultimately improving the overall outcomes of IVF/ICSI infertility treatment.

## 2. Materials and Methods

### Individuals selection

This cross-sectional study involved 734 individuals, encompassing 1000 IVF/ICSI cycles, conducted at 2 infertility centers (Milad and Novin) in Mashhad, Iran from 2016-2017. The participating centers comprised a public institution affiliated with the University of Medical Sciences and a private infertility center, with inclusion criteria encompassing all infertile couples who completed their IVF/ICSI cycles in these facilities. Data for the study were extracted from the medical records of these 734 individuals.

In both infertility centers, IVF and ICSI are performed together. All infertile couples who completed their IVF/ICSI cycle from these centers were included in the study. Also, data from couples who need sperm or donor eggs, or surrogacy uterus were excluded. Only the first 3 cycles of treatment were considered. Also, all infertile couples who left their treatment cycle incomplete or missed more than 50% of the required clinical factors were excluded. This dataset includes 38 features or prediction factors. 317 cycles of these patients (31.7%) had a successful clinical pregnancy, and 683 cycles had an unsuccessful result (68.3%). Also, 258 cycles (25.8%) had a successful ongoing pregnancy, and 742 cycles (74.2%) had an unsuccessful result. IVF/ICSI dataset included women with a mean age of 30.9 yr and men with a mean age of 35.4 yr. About 80% of couples were in the first cycle of treatment.

### Outcome

Clinical pregnancy was defined as a customarily shaped intrauterine gestational sac on ultrasound approximately 4 wk after the insemination or at approximately 6 wk of gestation. Also, we defined ongoing pregnancy as the fetal heart rate (FHR) in the sac of pregnancy in the 11 ${\;}^{\text{th}}$ wk after fetal transfer. Our data included couples undergoing IVF/ICSI treatment, with 31.7% of successful clinical and 27.96% of ongoing pregnancy.

### Methodology

In this study, we propose a hybrid feature selection method aimed at identifying the most important features influencing the success rate of infertility treatment. This method combines both filter and embedded methods and selects the best model based on a novel scoring system called hybrid feature selection scoring system.

The proposed hybrid method consists of 5 steps, each contributing to the overall process of feature selection. Firstly, the dataset is divided into training and test data sets, with the training data accounting for 80% of the total dataset and the remaining 20% reserved for testing purposes (Step 1). Following the data split, the filter and embedded methods are applied to identify and eliminate low-importance features, thus reducing the dimensionality of the dataset (Step 2).

To determine the best filter/embedded methods, a scoring system based on HFSs is employed (Step 3). This scoring system helps evaluate the performance of different feature selection techniques and selects the one yielding the most promising results. Once the best model is determined, the selected features are incorporated into wrapper methods. The training data is utilized to train a random forest model (RFM), leveraging the chosen features (Step 4).

**Figure 1:**
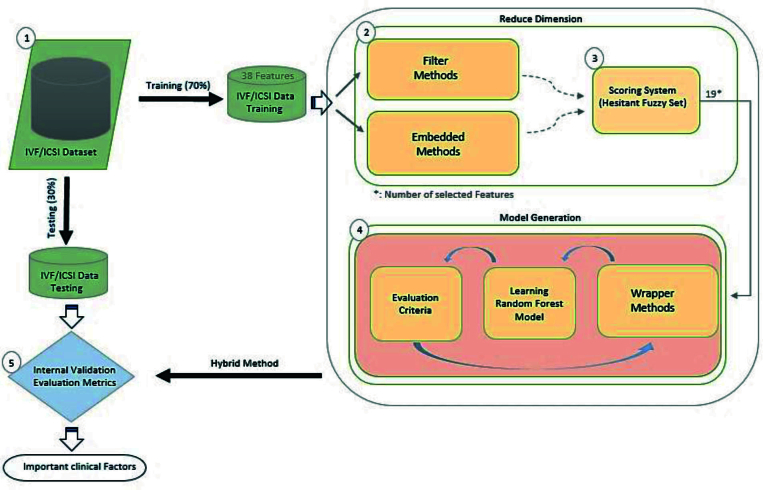
Graphical abstract of the study approach, IVF: In vitro fertilization, ICSI: Intra-cytoplasmic sperm injection.

Finally, the obtained hybrid methods are applied to the testing data and cross-validation techniques are employed for further refining the selection of essential features (Step 5). This step ensures that the final set of features is both robust and reliable in predicting the success rate of infertility treatment. For a visual representation of the proposed method and its study design, please refer to figure 1. By following this systematic approach, we aim to uncover the most influential features that contribute to the success rate of infertility treatment. The steps outlined in this methodology section demonstrate the necessity of each stage in the feature selection process, and how collectively they contribute to the effectiveness and reliability of our proposed hybrid method.

Herein, we used filter and embedded methods, including variance threshold (VT), L1-based selection (L1-based), k-Best selection (k-Best), and tree-based selection (tree-based) to reduce dimension. In addition, wrapper methods, such as sequential forward selection (SFS), sequential backward selection (SBS), sequential floating forward selection (SFFS), sequential floating backward selection (SFBS), and random selection applied in the modeling step to select principle features, which have recently been used in articles (17-21). Since wrapper methods use a machine learning model to evaluate their performance when selecting optimal features, an RFM is used. RFM is a robust ensemble method in infertility treatment that uses decision tree classifiers and majority votes to predict (22). Moreover, we included a detailed pseudo code along with a semi-flowchart to further enhance the clarity and understanding of our proposed method. For more information and a comprehensive visual representation, please refer to the supplementary file titled “Pseudo.docx".

#### Filter method

Filter methods are split into univariate and multivariate approaches (23). Herein, we focused on the univariate approach to improve feature selection methods. The VT is the statistical test and simple baseline approach in the univariate filter method. In this study, we considered threshold = 0.35, which obtains the highest accuracy (ACC) for VT. Another univariate approach is to select k-Best. The k-Best is a filter-based method that removes features in terms of ANOVA F-value between each feature and the target vector.

#### Embedded method

In these methods, the search for an optimal subset of features is performed during the modeling phase (24). L1-based feature selection (L1-based) is an embedded method that selects features as part of the model construction process. Moreover, tree-based is another popular embedded method that includes a forest of the tree to decide on removing features. A decision tree is a classifier built up using different splitting criteria (25).

#### Wrapper method

Wrapper-based feature selection methods have selected a subset of features and trained the model with each iteration. This process continues until the best subset is achieved based on the model evaluation function. Sequential feature selector methods are greedy search algorithms that try to eliminate redundant and irrelevant features by reducing the number of features and increasing the model's performance. We considered k = 7 as the number of features in the wrapper methods according to the expert idea for selecting a suitable number of features from 38.

### Evaluation metrics

Various measures can be used to evaluate the performance of different methods. Most applicable measures for evaluating the model include ACC and area under the receiver operating characteristic (ROC) curve (AUC), precision or positive predictive value (PPV), recall, and F-score. Considering the imbalanced dataset, criteria, such as ACC and recall, may not be decisive criteria because they are presented according to those couples with successful outcomes (true positive rate). In contrast, most infertile couples in our dataset have cycles with unsuccessful outcomes.

Therefore, we used Matthew's Correlation Coefficient (MCC) criterion, representing a robust criterion for evaluating model performance in both groups.

The MCC is used in machine learning to measure the quality of binary classifications, introduced by biochemist Brian W Matthews (26). This criterion considers positive and negative cycles and is considered a balanced criterion that can be used even if the classes are of very different sizes. MCC is a value of correlation coefficient between -1 and 1, in which 1 means a complete forecast and -1 shows an inverse forecast. MCC is calculated by the following equation: 


$$MCC=\frac{TP*TN-FN*FP}{\sqrt{\left(TP+FP)(TP+FN)(TN+FP)(TN+FN\right)}}$$

Where TP is the true positive value determined by the model, TN is the correct negative value, and FN and FP are, the negative and positive values the model incorrectly specifies. There are several measures applied for comparison, which are briefly specified below: 


$$Accuracy=\frac{TP+TN}{N}$$

$$PPV=\frac{TP}{TP+FP}$$

$$Recall=\frac{TP}{TP+FN}$$

$$F-Score=\frac{2*Recall*PPV}{Recall+PPV}$$

### Proposed hybrid method by HFS

Since problem-solving speed is high in filter/embedded methods, the runtime measure for comparing methods is not significant. We also noted that the purpose of using a filter/embedded method in the preprocessing phase is to remove low-significance features and gain a reduced dimension. Therefore, the number of features criterion does not matter at the phase. Therefore, we obtained the best filter-based method according to the 6 evaluation criteria in table I.

For this purpose, we applied an HFS. HFSs were presented as a generalization of simple fuzzy sets (27). HFSs are useful in medical decision-making when the expert hesitates between several values. This theory has been proven, to help enhance discernment in decision-making (28). We supposed that: 


$$A=\left\{<x.h_{A}\left(x\right)>|x\in X\right\}$$

Where $h_{A}\left(x\right)=\{ACC.MCC.F-Score.AUC.$ Recall.PPV} and X={VT,Tree-based,L1- based,andk-Best} . So, for each method x in X, there are several values, shown as $h_{A}\left(x\right),$ to evaluate the method's performance, such as ACC, AUC, F-score, MCC, PPV, and recall. Because each method has different criteria for evaluation, we used the corresponding HFS. Also, the criteria values varied in the range of 0-1. Then, MCC was transferred to [0, 1] for each method to correct the comparison. So, for each method x , we have 


A={<x.{ACC.AUC.F−Scor.MCC.PPV.Recall}>∣x∈X)}

To decide on the best performance for each method, we used the scoring system of HFS provided by Liao and Xu (29) as follows: 


σ¯'(h)=1lh∑γi.γj∈h(γi−γj)2

Where $l_{h}$ and $\gamma_{i}$ are the number and values of elements in $h_{A}(x)$ , respectively. In other words, σ¯'(h) is called the deviation degree of $h_{A}(x)$ , which reflects the standard deviation among all pairs of elements in the HFS. Therefore, we considered the function SF(h) in terms of σ¯'(h) as follows: 


SFh=1σ¯'h

So, SF(h) is called the scoring function (SF) of $h_{A}(x)$ . This function denoted the score of each method x.

**Table 1:** Results of filter/embedded methods on random forest classifier using IVF/ICSI dataset


**Filter /embedded methods**	**ACC**	**Runtime (s)**	**NFS**	**AUC**	**MCC-(normalized value)**	**F-score**	**PPV**	**Recall**
**VT**	0.681	2.17	9	0.55	0.159-(0.57)	0.62	0.65	0.68
**k-Best**	0.786	0.617	19	0.77	0.487-(0.743)	0.77	0.79	0.79
**L1-based**	0.781	0.931	15	0.74	0.474-(0.737)	0.76	0.79	0.78
**Tree-based**	0.79	1.509	20	0.75	0.5-(0.75)	0.77	0.79	0.8
IVF: In vitro fertilization, ICSI: Intracytoplasmic sperm injection, ACC: Accuracy, NFS: Number of feature selection, AUC: Area under the ROC curve, MCC: Matthew's correlation coefficient, PPV: Positive predictive value, VT: Variance threshold, L1-Based: L1-based selection, k-Best: k-Best selection, Tree-based: Tree-based selection								

### Ethical considerations

This study was approved by the Institutional Review Board of Mashhad University of Medical Sciences, Mashhad, Iran (Code: IR.MUMS.MEDICAL.REC.1399.060). According to the Helsinki Declaration, we have complied with the subject's ethics. We have obtained informed and free written consent from the participants, to apply their data in all infertility projects.

### Statistical analysis

These results were obtained using Python software version 3.8, which was implemented on a system equipped with 2 GB of RAM and a Core i3 CPU, enhancing the accuracy and efficiency of the analysis. Furthermore, for statistical analysis, IBMⓇ SPSSⓇ (Statistical Package for the Social Sciences) version 25 was employed, ensuring robust and comprehensive data processing.

## 3. Results

First, we implemented VT, tree-based, L1-based, and k-Best for IVF/ICSI dataset. The threshold limit in the variance method is 0.35, which provides the best ACC. The results of the filter-based methods using a random forest algorithm are given in table I. This table also presents influential factors selected after treatment.

We selected the RFM regarding its best results among well-known machine learning models for dataset (30).

The goal of feature selection is choosing the minimum feature and the highest performance of the model. The tree-based method has the best ACC (0.79) among filter-based methods, although it has more features (k = 20) than the other methods. Also, for this method, MCC = 0.5 indicates the correct performance of the model for selecting features. With a slight difference, the k-Best method has obtained a relatively good ACC (0.786) with MCC = 0.487 and 19 features selected. However, regarding the value of AUC, k-Best has a higher value (0.736) than tree-based with an AUC of 0.7 (Table I).

We also used 5 standard wrapper-based methods for feature selection using an RFM on IVF/ICSI dataset (Table II). This step has not used any preprocessing method. We noted that the random selection method obtains the best subset of features in different iterations based on ACC. Since the feature selection in this method is based on the model, we included it in wrapper methods. We validated models by k-fold cross-validation with k = 10.

**Figure 2:**
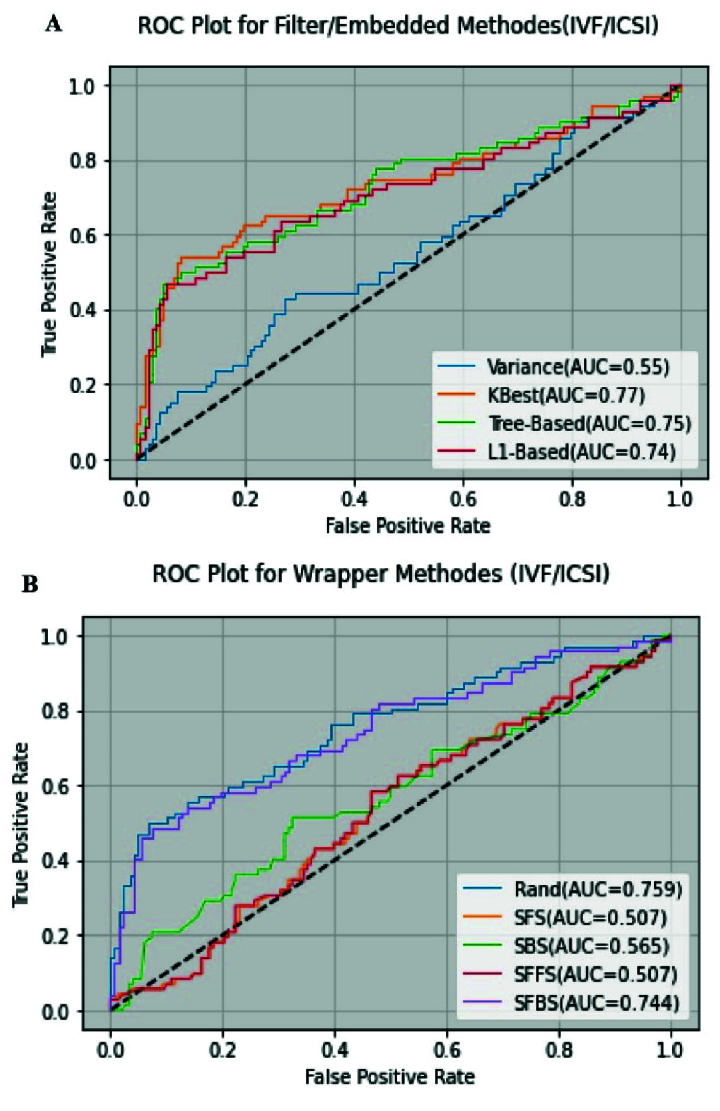
A) ROC curve for filter/embedded methods, B) ROC curve for wrapper methods in IVF/ICSI data set. ROC: Receiver operating curve, IVF: In virto fertilization, ICSI: Intra-cytoplasmic sperm injection, SFS: Sequential forward selection, SBS: Sequential backward selection, SFFS: Sequential floating forward selection, SFBS: Sequential floating backward selection, RS: Random selection, VT: Variance threshold, L1-based: L1-based selection, k-Best: K-Best selection, Tree-based: Tree-based selection.

According to the results of table II, a random search method with the best ACC (0.786) and higher AUC (0.759) is obtained. Figures 2 (A) and 2 (B) show the ROC curve for filter/embedded and wrapper methods using random forest algorithms.

These methods (filter, embedded, and wrapper) did not obtain feature selection goals, namely higher ACC and fewer features alone. Therefore, these methods were combined to develop a hybrid model to select practical features.

Then, values of the SF for each HFS in filter/embedded-based methods are calculated as follows:

As shown in table III, the k-best method has the highest SF (42.735). Therefore, we used its results for applying to the modeling stage.

Since the tree-based method has the second-highest SF in embedded methods, we used the mixed results of the tree-based method in the final comparison.

**Figure 3:**
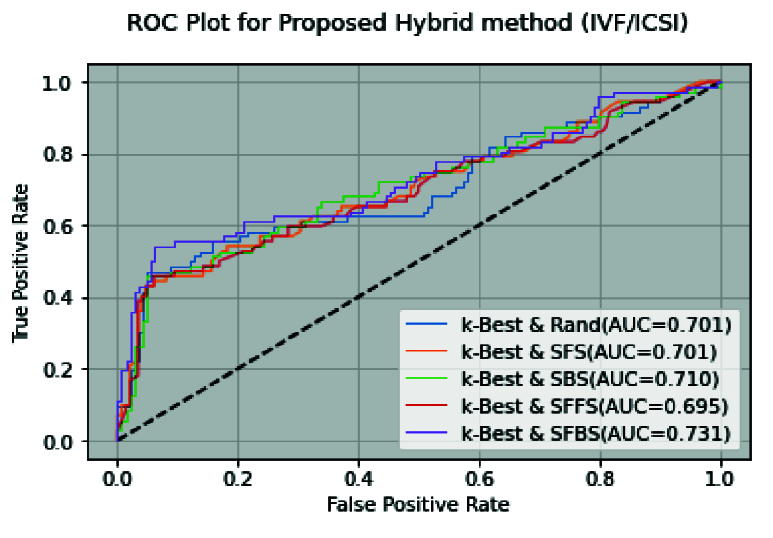
ROC curve for wrapper methods using k-Best (IVF/ICSI), ROC: Receiver operating characteristic, AUC: Area under the ROC curve.

First, among the filter/embedded-based methods, the k-Best method is considered for preprocessing stage. Then, we applied 19 features selected by the k-Best method for wrapper methods in the modeling stage. The k-Best and SFS method showed that this hybrid method increases the model accuracy (0.754-0.795) and simultaneously reduces runtime (from 737 to 89s). This improvement can be seen in other criteria, such as MCC, F-score, PPV, and recall. Also, the number of selected features obtained is n = 7 (Table IV). Furthermore, figure 3 shows ROC curve hybrid wrapper-based methods.

### Statistical analysis

**Figure 4:**
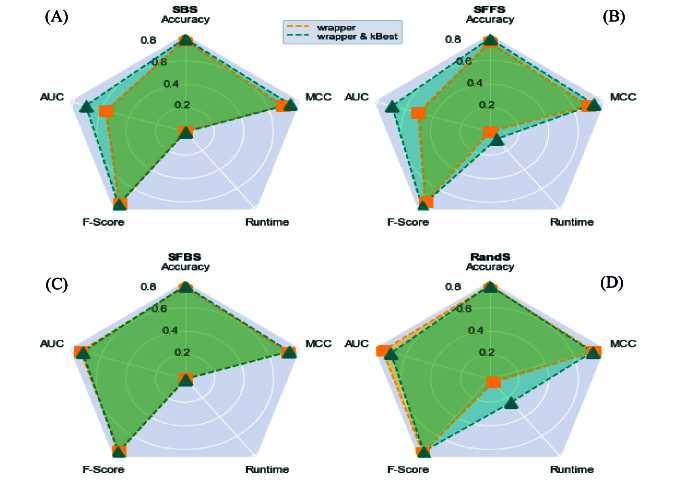
A) Spider plots to indicate impact of k-Best on SBS, B) Impact of k-Best on SFFS, C) Impact of k-Best on SFBS, D) Impact of k-Best on RandS.

This hybrid method aims to reduce the number of features and increase ACC, PPV, recall, AUC, MCC, and F-score values. We used spider plots to compare hybrid methods regarding the principle criteria. It is important to note that for the accurate comparison and considering that the short runtime indicates a better method, we have used the inverse of this value for this measure. Then, according to the result in figure 4, the larger area of the polygon formed by connecting the values of each criterion has a better performance of the model.

**Figure 5:**
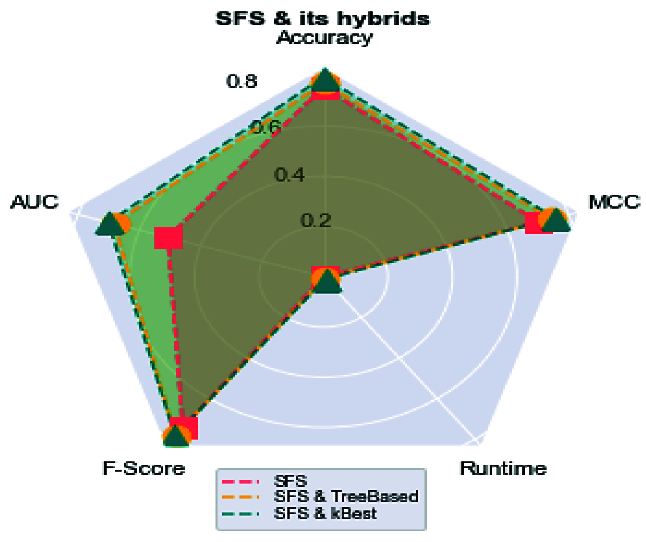
Spider plot for comparison among SFS, hybrid k-Best and SFS and hybrid tree-based and SFS methods.

In addition, the results obtained in figure 5 show that the hybrid SFS and k-Best method has achieved the best performance compared to the wrapper-based methods. In figure 5, a comparison of the improvement of the criteria presented for the SFS method before and after applying the k-Best and tree-based methods is presented. In this figure, the better method has more area based on the spider plot among the 3 proposed methods. As can be seen, the proposed hybrid method (SFS and k-Best) has a higher performance than the other 2 methods.

Table V shows the features selected to predict the success after treatment IVF/ICSI by each method. We used the get_metric_dict method of the panda's package in Python for the sequential feature selector object. This method displays the output of SFS as a data frame. The columns avg_score and ci_bound represent the average and confidence interval around the computed cross-validation scores (CI = 95%). Also, the columns std_dev and std_err represent the cross-validation scores' standard deviation and standard errors, respectively.

**Figure 6:**
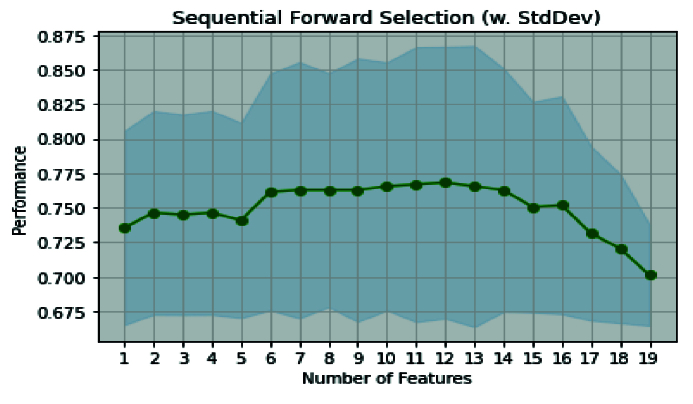
Comparison of performance of model based of feature's number.

In the complete set of features obtained by k-Best, 19 features were selected. The SFS and k-Best method obtained the best ACC for 7 features. Figure 6 shows the model's performance based on the ACC and the number of selected features. The ACC of the RFM without using feature selection was 0.76. After the proposed hybrid method, its ACC was improved to 0.795, as well the other criterias.

### Clinical analysis

**Figure 7:**
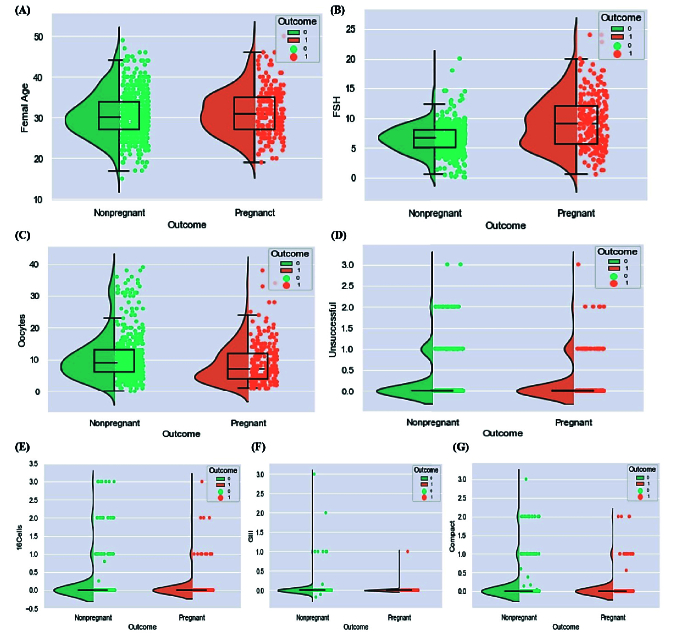
A) Relationship of female age and outcome of treatment, B) Relationship of dose 3 day FSH and outcome of treatment, C) Relationship of number of retrieved oocytes and outcome of treatment, D) Relationship of number of pre unsuccessful IVF/ICSI treatment and outcome, E): Relationship of number of cells day (16Cells) and outcome of treatment, F) Relationship of quality of transferred embryos (GIII) and outcome of treatment, G) Relationship of number of cells day (compact) and outcome of treatment, outcome of treatment, that is, FHR.

In our infertility dataset, the clinical pregnancy rate (CPR) is 32.87%, and FHR is 27.96%. The proposed hybrid method reduced practical features to 7 (from 38) principle features by SFS and k-Best hybrid method for clinical analysis. According to the results, day 3 follicle-stimulation hormone (FSH), number of cells day (16Cells), female age (FAge), number of oocytes collected (oocyte), quality of transferred embryos (GIII), number of cells day (compact), and number of pre unsuccessful IVF/ICSI (unsuccessful) have been reported as essential features of this method after treatment. Herein, we investigated the relationship between the principal factors and the pregnancy's success rate. Significate factors are obtained by Student *t* test. Since, statistical tests help determine the significance of observed differences or associations, we implemented Student *t* test for continuous value and Fisher's test for categorical variables to determine the significance of differences between means of 2 groups. The result showed that with increasing FAge, FHR, and CPR decreased. In addition, figure 7 (A) shows that the mean age in pregnant women is approximately 30 yr old, which has a significant difference from women who are not pregnant (p = 0.001). In addition, the highest FHR of 31.87%, is obtained for the FSH dose in 10-13 (mIU/ml). It can be seen in figure 7 (B) that the mean FSH dose in pregnant women is significantly lower than the rest (p = 0.001).

Moreover, the mean of oocytes collected in pregnant women was less than in another group. This difference was not significant based on FHR, and it had a significantly different base on CPR (p = 0.009) (Figure 7C). From the changes in CPR and ongoing pregnancy, it can be concluded that the highest success rate of pregnancy is obtained with < 6 retrieved oocytes. This amount decreases as the number of oocytes collected increases until the number of oocytes collected exceeds 29. Also, figure 7 (D) shows the relationship between the number of previous IVF/ICSI treatments and outcome. Although no significant difference was observed between successful and unsuccessful ongoing pregnancy groups for women who had a previous unsuccessful IVF/ICSI treatment; women who had 3 previous unsuccessful treatments are less likely to become pregnant. Although the risk of pregnancy decreases with increased number of unsuccessful treatments (3 or more), it can still be hoped that pregnancy is possible.

Similarly, the negative effect of features including 16Cells, GIII, and compact on the success of IVF/ICSI between 2 groups can be seen in figure 7 (E, F, G), respectively. The difference between ongoing pregnancy in the 2 groups is significant for compact factors. Also, a significant difference exists between successful clinical pregnancy and unsuccessful for 16Cells (p = 0.01) and GIII (p = 0.039).

**Figure 8:**
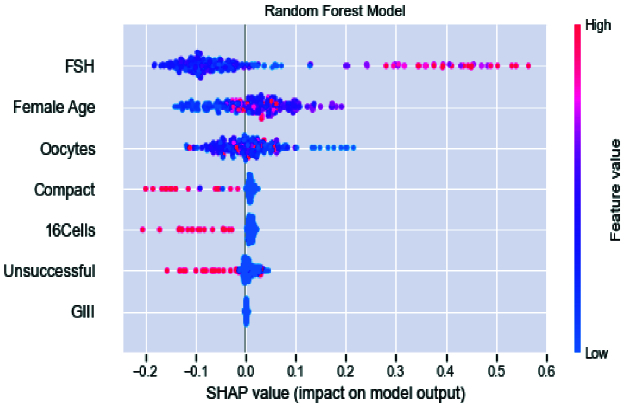
Shapley Additive exPlanations value for important clinical factors, output considered as FHR in RF model.

Finally, in figure 8, the shapley additive explanations value plot for principal clinical factors and those effects on pregnancy prediction are presented. As can be seen, for most patients, FSH factor, FAge, and oocytes have a negative relationship in predicting pregnancy with an impact factor of < 2. There were patients in whom the FSH factor was positively associated with predicting pregnancy rates (with an impact factor greater than 3). Also, there were patients who were FAge and oocytes positively impacted ( < 2) on the prediction. Almost all patients are divided into 2 categories based on unsuccessful, 16Cells, compact, and GIII. The first group has a high effect on the model prediction. It is negatively related to pregnancy with an impact factor of < 2. The rest have a positive relationship with pregnancy despite a low effect on the model prediction. It is indicated that these clinical factors are almost inversely related to the pregnancy outcome and behave independently of other features. Although factors such as FAge were negatively associated with pregnancy, many patients became pregnant despite advanced age. It can be concluded that other clinical factors affect these features, which may change treatment outcomes.

**Figure 9:**
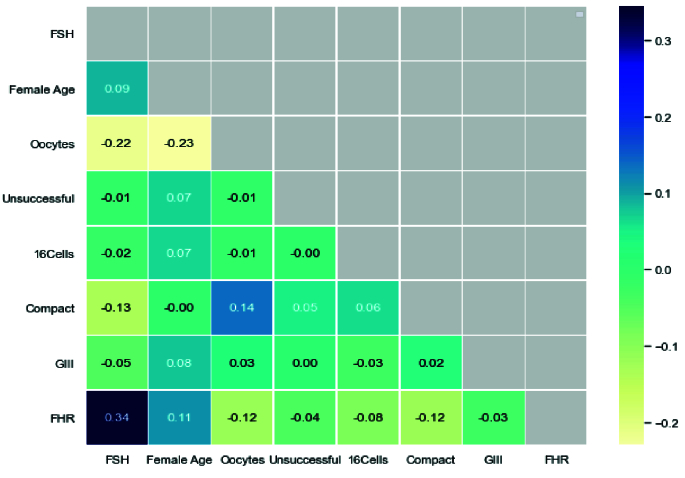
Heat map indicate correlation among features and outcome, outcome considered as FHR.

We also used a heat map to show the relationship between the selected features and the model's output (pregnancy and non-pregnancy). This map shows the correlation between the 2 features based on the Pearson function with a threshold of 0.85. As shown in figure 9, the ongoing pregnancy is positively correlated with features, including FAge (0.11) and FSH (0.34), and negatively related with oocytes (0.12), unsuccessful (0.04), 16Cells (0.08), compact (0.12), and GIII (0.03).

**Figure 10:**
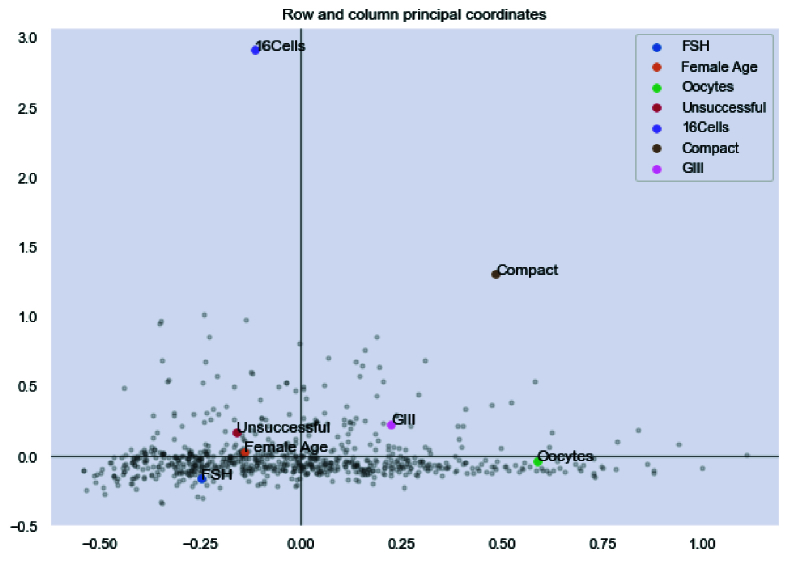
Association of selected features base on hybrid model.

Finally, we analyzed the efficacy of the proposed model by multiplecorrespondence analysis, which allows the investigation of the association between 2 or more qualitative variables. The result showed that most of the selected features lay in -0.5 to 0.5 intervals, demonstrating the proposed model's efficacy (Figure 10).

Furthermore, we enhanced the methodology section by including additional numerical analysis, which highlights the association among clinical factors and the feature of interest. These detailed numerical analyses can be found in the supplementary file titled “Feature_Association.docx". By presenting this comprehensive analysis, we aim to provide a thorough understanding of the relationship between clinical factors and the feature under investigation.

**Table 2:** Results of wrapper methods on random forest classifier using IVF/ICSI dataset


**Wrapper methods**	**ACC**	**Runtime (s)**	**NFS**	**AUC**	**MCC-(normalized value)**	**F-score**	**PPV**	**Recall**
**SFS**	0.754	737	7	0.507	0.405-(0.702)	0.74	0.75	0.75
**SBS**	0.777	3908	7	0.565	0.462-(0.731)	0.76	0.77	0.78
**SFFS**	0.754	2460	7	0.507	0.405-(0.702)	0.74	0.75	0.75
**SFBS**	0.777	12300	7	0.744	0.462-(0.731)	0.76	0.78	0.78
**RS **	0.786	240	14	0.759	0.48-(0.74)	0.77	0.79	0.79
IVF: In vitro fertilization, ICSI: Intracytoplasmic sperm injection, ACC: Accuracy, NFS: Number of feature selection, AUC: Area under the ROC curve, MCC: Matthew's correlation coefficient, PPV: Positive predictive value, SFS: Sequential forward selection, SBS: Sequential backward selection, SFFS: Sequential floating forward selection, SFBS: Sequential floating backward selection, RS: Random selection								

**Table 3:** Hybrid feature selection scoring system


**Method**	**VT**	**k-Best selection**	**L1-based**	**Tree-based**
**FS**	13.947	42.735	34.482	35.842
FS: Feature selection, VT: Variance threshold, L1-based: L1-based selection, k-Best: K-Best selection, Tree-based: Tree-based selection				

**Table 4:** Results of hybrid k-Best selection and wrapper methods on random forest classifier using IVF/ICSI dataset


**Hybrid k-Best** **and wrapper methods**	**ACC**	**Runtime (s)**	**NFS**	**AUC**	**MCC-(normalized value)**	**F-score**	**Recall**	**PPV**
**k-Best and SFS**	0.795	89	7	0.701	0.511 (0.755)	0.78	0.80	0.79
**k-Best and SBS**	0.790	158	7	0.710	0.500 (0.750)	0.77	0.79	0.80
**k-Best and SFFS**	0.786	123	7	0.695	0.489 (0.744)	0.78	0.79	0.78
**k-Best and SFBS**	0.786	162	7	0.731	0.487 (0.743)	0.77	0.79	0.79
**k-Best and RS**	0.786	42	8	0.701	0.462 (0.731)	0.76	0.78	0.77
IVF: In vitro fertilization, ICSI: Intracytoplasmic sperm injection, ACC: Accuracy, NFS: Number of feature selection, AUC: Area under the ROC curve, MCC: Matthew's correlation coefficient, PPV: Positive predictive value, SFS: Sequential forward selection, SBS: Sequential backward selection, SFFS: Sequential floating forward selection, SFBS: Sequential floating backward selection, RS: Random selection								

**Table 5:** Principle factors in detail with features selected by SFS and k-Best method for prediction in IVF/ICSI treatment


**Avg_score**	**Ci_bound**	**feature_idx**	**Feature_names**	**Std_dev**	**Std_err**
**0.735426**	0.112585	(1)	(FSH)	0.0702348	0.0405501
**0.746347**	0.118194	(1, 11)	(FSH, 16Cells)	0.0737338	0.0425702
**0.744981**	0.116388	(1, 11, 17)	(FSH, 16Cells, GIII)	0.0726071	0.0419197
**0.746355**	0.118291	(1, 11, 14, 17)	(FSH, 16Cells, Compact, GIII)	0.0737942	0.0426051
**0.74089**	0.113208	(1, 6, 11, 14, 17)	(FSH, Oocytes, 16Cells, Compact, GIII)	0.0706236	0.0407746
**0.761374**	0.137403	(1, 3, 6, 11, 14, 17)	(FSH, FAge, Oocytes, 16Cells, Compact, GIII)	0.0857171	0.0494888
**0.762755**	0.148693	(1, 3, 6, 8, 11, 14, 17)	(FSH, FAge, Oocytes, Unsuccessful, 16Cells, Compact, GIII)	0.0927602	0.0535551
SFS: Sequential forward selection, IVF: In vitro fertilization, ICSI: Intracytoplasmic sperm injection, Ave_score: Average_sacore, Ci_bound: Confidence interval, Feature_idx: Feature index, Std_dev: Standard deviation, Str_err: Standard error, FSH: Follicle-stimulation hormone					

## 4. Discussion

In infertility success prediction models, model accuracy is an important criteria that can be used to evaluate model performance. Also, we considered other criteria, such as ACC, recall, AUC, and F-score. The MCC criteria was used in this study due to the imbalance in the data studied. To compare the performance of the models, runtime was used according to the dimension of the problem. In deciding to choose the best dimensionality reduction method, we used HFSs and found that these sets can be more effective in choosing a hybrid method. The results showed that the method with lower ACC could obtain a model with high ACC and low specificity in combination with other methods.

Finally, according to the selected clinical features, we found that some useful features in predicting the model, although negatively related to pregnancy outcome, can have a different effect on pregnancy outcome by changing other clinical factors.

Different feature selection methods can be seen in various research. The SFFS was used for the feature selection method and applied the result to SVM (support vector machine), decision tree, and artificial neural network learning methods (31). Besides, a study obtained several essential features from their method based on the Chi-square test filter and *t* test statistic and applied them to the artificial neural network model (32). Moreover, it has used the evaluation criteria of OR, PPV, NPV, and p-value models for the IVF/ICSI infertility dataset. In another study the hill-climbing algorithm was used to select the features. They examined the performance of the SVM, RF, C4.5, MLP (multi-layer perceptron), and Cart models by selecting 25 features and using AUC, ACC, and F-score criteria (33). Recently, Kothandaraman et al. proposed new algorithm for ranking features and focuses on predicting the outcome of assisted reproductive technology using a dynamic model called ensemble of heterogeneous incremental classifier (EHIC) in machine learning. They introduce a feature ranking algorithm called voted information gain attribute rank estimation algorithm (VIGAREA) to enhance the performance of EHIC (34). Our proposed hybrid feature selection method aims to identify influential features in predicting the success rate of infertility treatment. We combined filter and embedded methods and selected the best model using the hybrid feature selection scoring system (HFSs) and the RFM. On the other hand, the EHIC with VIGAREA approach focuses on predicting the outcome of assisted reproductive technology using an ensemble of classifiers and the VIGAREA feature ranking algorithm. While both methods aim to improve infertility treatment outcome prediction, they differ in their specific techniques and approaches.

Our proposed algorithm utilizes HFS (hybrid feature selection), which incorporates the standard deviation among various criteria, enhancing the quality of feature selection. Additionally, we employ wrapper methods to further refine the feature selection process. The RFM is then applied to classify the success of infertility treatments. The results of our study demonstrate that the selected features effectively predict the success rate of infertility treatment.

### Limitations and strengths

This study has some limitations and precautions. The data were collected from only 2 infertility centers in one city. This limited scope may affect the generalizability of the findings. Future research should aim to collect data from multiple centers across different geographical locations to enhance the representativeness and external validity of the results. In addition, the size of the dataset used, particularly in the successful samples, was not significant. This imbalance may introduce bias and affect the statistical power of the analysis. It is important to consider this limitation when interpreting the results and to strive for larger and more balanced datasets in future studies. The suggested hybrid method selects the best model based on the novel approach in using the scoring system of HFS, which is considered an advantage of the study. Although the proposed hybrid method incorporates a novel approach using the scoring system of HFS, the study only employed a subset of standard feature selection methods. Exploring additional feature selection techniques and comparing their performance could provide further insights and enhance the robustness of the methodology.

The proposed hybrid method, which selects the best model based on the innovative scoring system of HFS, is a strength of the study. This approach enhances the accuracy and reliability of feature selection and contributes to the advancement of the field.

Furthermore, the research incorporates newer tools, such as the MCC measure, to assess model performance in unbalanced datasets. This demonstrates a thorough evaluation of the proposed method's effectiveness and ensures its suitability for practical applications.

Future studies could explore the application of heuristic algorithms for dimensional reduction and feature ranking. This could offer alternative approaches to selecting influential features and improve the overall methodology. In addition, external datasets can be utilized to evaluate and generalize the proposed model. This external validation would provide further confidence in the method's performance and reliability.

## 5. Conclusion

Our study introduces an innovative approach that leverages HFSs in feature selection, utilizing a multicenter dataset to predict infertility success rates. By considering the standard deviation among various criteria, HFSs improve feature selection quality and reduce feature quantity. Notably, our findings reveal significant distinctions in mean values between pregnant and non-pregnant groups for key features, including FSH, Age, 16Cells, oocytes, GIII, and compact. Additionally, we establish a noteworthy correlation between age and FHR and the CPR, with the highest FSH level (31.87%) observed within the FSH dose range of 10-13 (mIU/ml).

##  Conflict of Interest

The authors declare that there is no conflict of interest.
